# Association between oronasopharyngeal abnormalities and malocclusion in Northeastern Brazilian preschoolers

**DOI:** 10.1590/2177-6709.21.3.039-045.oar

**Published:** 2016

**Authors:** Genara Brum Gomes, Raquel Gonçalves Vieira-Andrade, Raulison Vieira de Sousa, Ramon Targino Firmino, Saul Martins Paiva, Leandro Silva Marques, Ana Flávia Granville-Garcia

**Affiliations:** 1 PhD student, Universidade Federal de Minas Gerais (UFMG), Department of Pediatric Dentistry and Orthodontics, School of Dentistry, Belo Horizonte, Brazil.; 2 PhD student, Universidade Federal de Pernambuco (UFPE), Department of Dentistry, School of Dentistry, Recife, Pernambuco, Brazil.; 3 Full Professor, Universidade Federal de Minas Gerais (UFMG), Department of Pediatric Dentistry and Orthodontics, School of Dentistry, Belo Horizonte, Brazil.; 4 Associate Professor, Universidade Federal dos Vales do Jequitinhonha e Mucuri (UFVJM), Department of Orthodontics, School of Dentistry, Diamantina, Minas Gerais, Brazil.; 5 Associate Professor, Universidade Estadual da Paraíba (UEPB), Department of Dentistry, School of Dentistry, Campina Grande, Paraíba, Brazil.

**Keywords:** Primary teeth, Epidemiology, Malocclusion, Preschool child

## Abstract

**Objective:**

Evidence is contradictory regarding the association between oronasopharyngeal abnormalities and malocclusion. The aim of the present study was to assess the association between oronasopharyngeal abnormalities and malocclusion (anterior open bite and posterior crossbite) in preschoolers.

**Methods:**

A cross-sectional study was conducted with a representative sample of 732 preschoolers aged 3-5 years old selected randomly from private and public preschools. Anterior open bite (AOB) and posterior crossbite (PC) were evaluated through a clinical exam. Parents/caregivers answered a questionnaire addressing sociodemographic indicators and oronasopharyngeal issues. Statistical analysis involved descriptive analysis and Poisson regression (*p* < 0.05).

**Results:**

The prevalences of AOB and PC were 21.0% and 11.6%, respectively. Being three years old (PR = 1.244; 95% CI = 1.110-1.394; *p* < 0.001), being four years old (PR = 1.144; 95% CI = 1.110 - 1.394; *p* = 0.015), absence of allergy (PR = 1.158; 95% CI = 1.057 - 1.269; *p* = 0.002), not having undergone nose surgery (PR = 1.152; 95% CI = 1.041 - 1.275; *p* = 0.006) and having a sore throat more than five times in the same year (PR = 1.118; 95% CI = 1.011 - 1.237; *p* = 0.030) were significantly associated with AOB. The absence of asthma (PR = 1.082; 95% CI = 1.012 - 1.156; *p* = 0.020), not having undergone throat surgery (PR = 1.112; 95% CI = 1.068 - 1.158; *p* < 0.001) and not having undergone nose surgery (PR = 1.114; 95% CI = 1.069 - 1.160; *p* < 0.001) remained associated with PC.

**Conclusion:**

Significant associations were found between oronasopharyngeal-reported abnormalities and the presence of AOB and PC in preschoolers.

## resumo

**Introdução:**

não há consenso na literatura quanto à associação entre alterações oronasofaríngeas e as más oclusões.

**Objetivo:**

o objetivo do presente estudo foi avaliar a associação entre as alterações oronasofaríngeas e as más oclusões (mordida aberta anterior e mordida cruzada posterior) em pré-escolares.

**Métodos:**

um estudo transversal foi conduzido com uma amostra representativa de 732 pré-escolares com 3 a 5 anos de idade, aleatoriamente selecionados em pré-escolas privadas e públicas. A mordida aberta anterior (MAA) e a mordida cruzada posterior (MCP) foram avaliadas a partir de exames clínicos. Pais/cuidadores responderam a um questionário relativo a indicadores sociodemográficos e problemas oronasofaríngeos. A análise estatística envolveu análise descritiva e regressão de Poisson (*p* < 0,05).

**Resultados:**

as prevalências de MAA e MCP foram de 21,0% e 11,6%, respectivamente. Ter três anos de idade (RP = 1,244; IC = 1,110 - 1,394; *p* < 0,001, considerando-se RP = Razões de Prevalência e IC = Intervalo de Confiança 95%), ter quatro anos de idade (RP = 1,144; IC = 1,110 - 1,394; *p* = 0,015), ausência de alergia (RP = 1,158; IC = 1,057 - 1,269; *p* = 0,002), não ter se submetido a cirurgias do nariz (RP = 1,152; IC = 1,041 - 1,275; *p* = 0,006) e ter tido dor de garganta mais de cinco vezes no mesmo ano (RP = 1,118; IC = 1,011 - 1,237; *p* = 0,030) foram significativamente associados com MAA. Ausência de asma (RP = 1,082; IC = 1,012 - 1,156; *p* = 0,020), não ter realizado adenoidectomia (RP = 1,112; IC = 1,068 - 1,158; *p* < 0,001) e não ter se submetido a cirurgias do nariz (RP = 1,114; IC = 1,069 - 1,160; *p* < 0,001) permaneceram associados à MCP.

**Conclusão:**

associações significativas foram encontradas entre as alterações oronasofaríngeas relatadas e a presença de MAA e MCP em pré-escolares.

## INTRODUCTION

Anterior open bite (AOB) and posterior crossbite (PC) are the most common types of malocclusion found in the primary dentition, with prevalence rates of about 46.2 % and 18.2%, respectively.^1^ ^,^ ^2^ These conditions have a multifactorial etiology that includes both genetic and acquired aspects.^3^ Acquired aspects included non-nutritive sucking habits, such as pacifier use and thumb sucking, as well as oronasopharyngeal abnormalities (hypertrophy of adenoids/tonsils, deviated septum, nasal obstruction, sinusitis, allergic rhinitis, bronchitis and asthma).^4^ ^,^ ^5^

The literature has shown that the presence of oronasopharyngeal abnormalities may contribute to changes in one's breathing pattern (nasal to mouth), which alters the balance between the tongue and facial musculature/soft tissues, thereby influencing the development of the skull.^6^ ^,^ ^7^ This may lead to disharmony in the growth of orofacial structures, such as maxillary constrictions, higher palatal vault and increased gonial angle, contributing to occlusal changes.^6^ ^-^ ^9^ However, evidence is conflicting. While some authors have found associations between AOB or PC and mouth breathing, allergic rhinitis, asthma and enlarged tonsils/adenoids^3^ ^,^ ^10^ ^,^ ^11^ ^,^ ^12^, others have not.^9^ ^,^ ^13^ ^,^ ^14^

The early identification of pharyngeal factors that predispose individuals to malocclusion in the primary dentition phase is of fundamental importance, as it may assist in determining an intervention at the appropriate age (before growth spurt), thereby allowing early normalization of respiration and both postural and dentoskeletal abnormalities.^15^ ^,^ ^16^

Population-based studies conducted with preschool children have found positive associations between oronasopharyngeal factors and malocclusion,^3^ ^,^ ^11^ ^,^ ^12^ ^,^ ^17^ but they have focused on evaluating specific problems, such as mouth breathing, atypical swallowing and allergic rhinitis. A population-based study has specifically evaluated the role of other abnormalities, such as hypertrophied adenoids/tonsils, in the etiology of malocclusion, failing to find an association.^13^ Thus, additional research is still necessary to clarify this issue.

The aim of the present population-based study was to evaluate the possible association between oronasopharyngeal abnormalities and malocclusion (AOB and PC) in the primary dentition of a randomized representative sample of preschool children.

## MATERIAL AND METHODS 

The present study was conducted in accordance with the Declaration of Helsinki and was independently reviewed and approved by a Human Ethics Research Committee (00460133000-11). A cross-sectional study was carried out involving 732 male and female children, aged between three and five years old, enrolled at 33 private and public preschools in the city of Campina Grande, Brazil. Participants were selected from a total population of 12,705 children at this age group (corresponding to 6.6% of the population). 

A two-stage sampling method was used to ensure representativeness. Preschools were randomly selected from each health district in the first stage, and children were randomly selected from each preschool in the second stage. A total of 18 of the 127 public preschools and 15 of the 122 private preschools in the city were randomly selected. Sample size was calculated based on data acquired in a pilot study, considering a 4% margin of error, a 95% confidence level and a 50.0% prevalence rate of malocclusion. A correction factor of 1.2 was applied to compensate for the design effect. The minimum sample size was estimated at 720 schoolchildren, which was deemed sufficient for a power of 80%. A further 20% was added to compensate for potential losses, giving a total sample of 864 schoolchildren.

Inclusion criteria were as follows: age between three and five years old; enrolment in a preschool; absence of systemic disease (according to parents'/caregivers' reports); exclusively in the primary dentition phase; all primary teeth present; no loss of mesiodistal diameter due to dental caries; no history of orthodontic treatment; the return of the questionnaires; and cooperation during clinical examination. 

The calibration exercise consisted of two stages. The theoretical stage involved a discussion of the diagnostic criteria for AOB and PC and an analysis of photographs. An orthodontist was the gold standard in the theoretical framework and coordinated this step, instructing three general dentists on how to perform the examination. The second stage involved clinical examinations of randomly selected preschool children that were not part of the main sample. Dentists examined 50 children in the age bracket under study (3 to 5 years old). Interexaminer agreement was tested by comparing each examiner with the gold standard. Children were reexamined after a seven-day interval for determination of intraexaminer agreement. Cohen's Kappa coefficients were calculated (K = 0.85 to 0.90 for both interexaminer and intraexaminer agreement). As the Kappa coefficients were satisfactory, examiners were considered able to perform the epidemiological study.

A pilot study was conducted to calculate sample size and test the methodology and comprehension of the questionnaire on the part of parents/caregivers of 40 children who were not included in the main sample. Results revealed no misunderstandings regarding the questionnaire or any need to make changes in the method.

Parents/caregivers answered a questionnaire addressing sociodemographic data (child's age and sex, mother's schooling, monthly income, number of residents at home, number of siblings and type of preschool) and oronasopharyngeal factors (asthma, allergy, sinusitis, bronchitis, history of throat surgery, history of nose surgery, stuffy nose and history of sore throat). Monthly household income was categorized based on minimum wage in Brazil, which was equal to US$ 301.70 at the time. 

Clinical examination was performed after the return of the questionnaires. Oral examinations were performed by three dentists who were blinded to the responses on the questionnaires. Prior to examination, examiners brushed and flossed the children's teeth to remove bacterial biofilm (plaque) from the tooth surfaces. To this end, the children received a kit containing a toothbrush, fluoridated toothpaste and dental floss. Examinations were performed by the dentists at the preschools who were in knee-to-knee position, with the aid of a portable lamp attached to the examiner's head (Tikkina 2, Petzl, Rawang, Malasya). Individual cross-infection protection equipment was used. Packaged and sterilized mouth mirrors (PRISMA^TM^, São Paulo, SP, Brazil), OMS probes (Golgran Ind. e Com. Ltda., São Paulo, SP, Brazil) and dental gauze (used to dry the teeth) were used for examination.

During clinical examination, aspects of AOB and PC were recorded. No radiographs were used for diagnosis.^18^ ^,^ ^19^ The absence of a vertical overlap of maxillary incisors in relation to mandibular incisors was recorded as AOB.^18^ PC was recorded when maxillary molars occluded in lingual relationship with mandibular molars in centric occlusion.^19^

Descriptive and analytical statistics were performed considering a 5% level of significance (*p* < 0.05). Descriptive statistics were used for characterization of the sample and determination of the prevalence of AOB and PC. Two bivariate Poisson regression models were constructed: one for each type of malocclusion. Sociodemographic indicators and oronasopharyngeal factors were the independent variables. Poisson multivariate analysis was performed for AOB and PC. Independent variables with a *p*-value < 0.20 in the bivariate analysis were incorporated into the multivariate models using the forward stepwise method. Variables with a *p*-value < 0.05 were kept in the final models. Adjusted and unadjusted prevalence ratios (PR) and 95% confidence intervals (CI) were calculated. Data organization and statistical analyses were carried out by means of Statistical Package for Social Science (SPSS for Windows, version 18.0, SPSS Inc, Chicago, IL, USA).

## RESULTS 

The sample consisted of 732 children aged between three and five years old (mean: 46.87 ± 8.70 months), corresponding to a response rate of 84.72 % of the total selected, based on sample size calculation (n = 864). Losses (15.28%) were due to absence from preschool over three times on the days scheduled for clinical examination (n = 76), in addition to lack of cooperation during examination (n = 56). Most children examined were boys (52.5%), studied at a public preschool (51.8%) and had parents/caregivers who were 30 old or younger (51.3%). Considering sociodemographic variables, the majority of parents/caregivers (80.2%) reported a household income lower than or equal to three times the Brazilian minimum wage, and most mothers had more than eight years of schooling (56.1%). The prevalence of AOB was 21.0% and the prevalence of PC was 11.6%, which was more frequent among children aged 48 months (4 years old). 

Table 1 displays the associations between AOB and the independent variables. In the adjusted multivariate regression (Poisson regression), being three years old, being four years old , absence of allergy , not having undergone nose surgery and having a sore throat more than five times in the previous 12 months remained significantly associated with AOB, regardless of the other variables.

**Table 1:** Poisson bivariate analysis and multivariate regression model for anterior open bite (AOB) and independent variables among preschool children aged between three and five years old (n = 732).

Variables	AOB		Bivariate Unadjusted prevalence ratio *		Multivariate Adjusted prevalence ratio ^†^		
	Present	Absent					
	n (%)	n (%)	*p*-value	(95% CI)	*p*-value	(95% CI)
Sex						
Female	76 (21.8)	272 (78.2)			-	-
Male	78 (20.3)	306 (79.7)			-	-
Child's age (years)						
3	62 (27.0)	168 (73.0)	< 0.001	1.148 (1.079-1.222)	< 0.001	1.244 (1.110-1.394)
4	75 (22.0)	266 (78.0)	0.001	1.103 (1.043-1.167)	0.015	1.144 (1.027-1.274)
5	17 (10.6)	144 (89.4)		1.00		1.00
Mother's schooling^1^						
≤ 8 years of study	91 (28.3)	230 (71.7)	< 0.001	1.113 (1.060-1.169)	-	-
> 8 years of study	63 (15.3)	348 (84.7)		1.00	-	-
Monthly income^2^						
≤ 3 times min. wage	141 (24.0)	446 (76.0)	< 0.001	1.138 (1.082-1.198)	-	-
> 3 times min. wage	13 (9.0)	132 (91.0)		1.00	-	-
Number of residents at home^2^						
≤ 5	120 (19.4)	497 (80.6)		1.00	-	-
≥ 6	33 (30.8)	74 (69.2)	0.013	1.095 (1.019-1.177)	-	-
Only child						
Yes	33 (13.6)	209 (86.4)		1.00	-	-
No	121 (24.7)	369 (75.3)	< 0.001	1.097 (1.045-1.152)	-	-
Type of preschool						
Public	104 (27.4)	275 (72.6)	< 0.001	1.116 (1.064-1.171)	-	-
Private	50 (14.2)	303 (85.8)		1.00	-	-
Birth weight^2^						
< 2500 g	16 (30.8)	36 (69.2)	0.890	1.090 (0.987-1.204)	-	-
≥ 2500 g	123 (20.0)	493 (80.0)		1.00	-	-
Child's health problems						
Respiratory	40 (20.5)	155 (79.5)		1.00	-	-
Other	10 (27.8)	26 (72.2)	0.354	1.060 (0.937-1.200)	-	-
Allergy						
Yes	21 (15.0)	119 (85.0)		1.00		1.00
No	30 (32.6)	62 (67.4)	0.002	1.153 (1.055-1.260)	0.002	1.158 (1.057-1.269)
Asthma						
Yes	14 (38.9)	22 (61.1)	0.014	1.168 (1.033-1.322)	-	-
No	37 (18.9)	159 (81.1)		1.00	-	-
Sinusitis						
Yes	5 (17.2)	24 (82.8)		1.00	-	-
No	46 (22.7)	157 (77.3)	0.483	1.046 (0.922-1.187)	-	-
Bronchitis						
Yes	3 (27.3)	8 (72.7)		1.00	-	-
No	48 (21.7)	173 (78.3)	0.679	1.046 (0.846-1.292)	-	-
History of throat surgery						
Yes	1 (12.5)	7 (87.5)		1.00	-	-
No	153 (21.3)	566 (78.7)	0.473	1.078 (0.878-1.324)	-	-
History of nose surgery						
Yes	1 (8.3)	11 (91.7)		1.00		1.00
No	152 (21.3)	562 (78.7)	0.131	1.120 (0.967-1.296)	0.006	1.152 (1.041-1.275)
Child always has stuffed up nose						
Yes	43 (26.5)	119 (73.5)	0.061	1.059 (0.997-1.125)	-	-
No	110 (19.5)	455 (80.5)		1.00	-	-
Child always keeps mouth open						
Yes	47 (30.5)	107 (69.5)	0.003	1.100 (1.034-1.170)	-	-
No	107 (18.7)	466 (81.3)		1.00	-	-
Child had sore throat more than 5 times in the previous 12 months						
Yes	42 (26.2)	118 (73.8)	0.082	1.055 (0.993-1.121)	0.030	1.118 (1.011-1.237)
No	112 (19.6)	458 (80.4)		1.00		1.00
Child had sinusitis in the previous 12 months						
Yes	21 (19.6)	86 (80.4)		1.00	-	-
No	132 (21.3)	489 (78.7)	0.698	1.014 (0.947-1.085)	-	-
Oronasopharyngeal abnormalities						
Yes	74 (22.8)	250 (77.2)	0.288	1.027 (0.978-1.079)	-	-
No	80 (19.6)	328 (80.4)		1.00	-	-

*  Unadjusted Poisson regression. ^†^ Poisson regression adjusted for independent variables and posterior crossbite.

1 Equivalent to elementary and middle school. ^2^ Categorized based on the median of the crude value.

Absence of asthma, not having undergone throat surgery and not having undergone nose surgery remained significantly associated with PC, regardless of the other variables (Table 2).

**Table 2:** Poisson bivariate analysis and multivariate regression model for posterior crossbite (PC) and independent variables among preschool children aged between three and five years old (n = 732).

Variables	PC			Bivariate Unadjusted prevalence ratio *		Multivariate Adjusted prevalence ratio ^†^	
	Present	Absent					
	n (%)	n (%)	*p*-value	(95% CI)	*p*-value	(95% CI)
Sex						
Female	50 (14.4)	298 (85.6)	0.027	1.048 (1.005-1.093)	-	-
Male	35 (9.1)	349 (90.9)		1.00	-	-
Child's age (years)						
3	26 (11.3)	204 (88.7)	0.522	1.018 (0.964-1.076)	-	-
4	44 (12.9)	297 (87.1)	0.222	1.033 (0.981-1.088	-	-
5	15 (9.3)	146 (90.7)		1.00	-	-
Mother's schooling^1^						
≤ 8 years of study	40 (12.5)	281 (87.5)	0.529	1.014 (0.972-1.057)	-	-
> 8 years of study	45 (10.9)	366 (89.1)		1.00	-	-
Monthly income^2^						
≤ 3 times min. wage	67 (11.4)	520 (88.6)		1.00	-	-
> 3 times min. wage	18 (12.4)	127 (87.6)	0.741	1.009 (0.957-1.064)	-	-
Number of residents at home						
≤ 5	71 (11.5)	546 (88.5)		1.00	-	-
≥ 6	13 (12.1)	94 (87.9)	0.850	1.006 (0.948-1.068)	-	-
Only child						
Yes	28 (11.6)	214 (88.4)		1.00	-	-
No	57 (11.6)	433 (88.4)	0.980	1.001 (0.957-1.046)	-	-
Type of preschool						
Public	44 (11.6)	335 (88.4)			-	-
Private	41 (11.6)	312 (88.4)			-	-
Birth weight^2^						
< 2500 g	5 (9.6)	47 (90.4)		1.00		
≥ 2500 g	74 (12.0)	542 (88.0)	0.580	1.022 (0.947-1.103)	-	-
Child's health problems						
Respiratory	16 (8.2)	179 (91.8)		1.00	-	-
Other	6 (16.7)	30 (83.3)	0.181	1.078 (0.966-1.204)	-	-
Allergy						
Yes	14 (10.0)	126 (90.0)	0.957	1.002 (0.933-1.076)		
No	9 (9.8)	83 (90.2)		1.00		
Asthma						
Yes	1 (2.8)	35 (97.2)		1.00		1.00
No	22 (11.2)	174 (88.8)	0.018	1.082 (1.013-1.156)	0.020	1.082 (1.012-1.156)
Sinusitis						
Yes	4 (13.8)	25 (86.2)	0.503	1.041 (0.926-1.169)	-	-
No	19 (9.4)	184 (90.6)		1.00	-	-
Bronchitis						
Yes	1 (9.1)	10 (90.9)	0.923	1.008 (0.859-1.183)	-	-
No	22 (10.0)	199 (90.0)		1.00	-	-
History of throat surgery						
Yes	0 (0.0)	8 (100.0)		1.00		1.00
No	85 (11.8)	634 (88.2)	<0.001	1.118 (1.095-1.142)	<0.001	1.112 (1.068-1.158)
History of nose surgery						
Yes	0 (0.0)	12 (100.0)		1.00		1.00
No	85 (11.9)	629 (88.1)	<0.001	1.119 (1.096-1.143)	<0.001	1.114 (1.069-1.160)
Child always has stuffed up nose						
Yes	19 (11.7)	143 (88.3)	0.987	1.000 (0.951-1.052)	-	-
No	66 (11.7)	499 (88.3)		1.00	-	-
Child always keeps mouth open						
Yes	20 (13.0)	134 (87.0)	0.584	1.015 (0.963-1.069)	-	-
No	65 (11.3)	508 (88.7)		1.00	-	-
Child had sore throat more than 5 times in the previous 12 months						
Yes	20 (12.5)	140 (87.5)	0.708	1.010 (0.959-1.063)	-	-
No	65 (11.4)	505 (88.6)		1.00	-	-
Child had sinusitis in the previous 12 months						
Yes	13 (12.1)	94 (87.9)	0.870	1.005 (0.947-1.067)	-	-
No	72 (11.6)	549 (88.4)		1.00	-	-
Oronasopharyngeal abnormalities						
Yes	38 (11.7)	286 (88.3)	0.930	1.002 (0.961-1.045)	-	-
No	47 (11.5)	361 (88.5)		1.00	-	-

*  Unadjusted Poisson regression. ^†^ Poisson regression adjusted for independent variables and anterior open bite.

1 Equivalent to elementary and middle school. ^2^ Categorized based on the median of the crude value.

## DISCUSSION 

Oronasopharyngeal abnormalities were significantly associated with both AOB and PC in the preschool children analyzed. However, comparisons with findings from previous studies are limited due to methodological differences and should therefore be interpreted with caution.^14^

Ages three and four years old were significantly associated with AOB. A similar finding is described in a cross-sectional study conducted in Nigeria,^20^ which reports an association between AOB and the age of four. Although no data on non-nutritive sucking habits were collected in the present study, such habits are common among Brazilian preschool children.^21^ It is possible that the reduction in these habits, which commonly occurs with the increase in age, contributed to the spontaneous self-correction of this type of malocclusion.^22^ The same line of reasoning did not apply to PC, perhaps because this type of malocclusion tends to be transferred from the primary to the permanent dentition.^18^

A recent systematic literature review considered at least three episodes of sore throat per year as the cutoff point to define recurrent sore throat;^23^ in the present study, parents/guardians were asked whether their children had throat or nose surgery and whether they had had a sore throat more than five times in the previous 12 months. This information was used as proxy variables for inflamed tonsils/hypertrophied adenoids. Hypertrophied adenoids are one of the major causes of upper airway obstruction,^24^ which can lead to mouth-breathing habit, thereby predisposing individuals to AOB.^12^ In agreement with these findings, significant associations were found in the present study between AOB and both "absence of nose surgery" and "having a sore throat more than five times in the previous 12 months." In contrast, Souki et al^14^ found no association between enlarged tonsils/adenoids and malocclusion. The authors state that genetic factors may explain why even a considerable air-flow obstruction may not be enough to cause occlusal alterations in children with low susceptibility to the development of malocclusion. Additional research is needed to clarify this issue.

Events such as recurrent allergies and asthma cause nasal obstructions and favor mouth breathing;^25^ they are therefore considered predisposing factors for malocclusion, such as AOB and PC, due to the effects produced on craniofacial growth in children.^26^ Opinions in the literature are divergent. While some authors have found an association between both asthma and allergic rhinitis and AOB,^11^ ^,^ ^27^ others have only found associations with PC.^3^ ^,^ ^10^ In the present study, however, "absence of allergy" was associated with AOB. It is possible that other factors served as confounding variables, such as pacifier use or the climate of the city of Campina Grande. Allergy and asthma are commonly found in locations with low temperatures.^28^ Campina Grande is located in Northeast Brazil where a hot, dry climate predominates. This may also explain the association between "absence of asthma" and PC. 

Chronic obstruction and an altered airway path can lead to the breakdown of normal functional breathing relationships.^29^ Not having undergone nose or throat surgery, which can lead to such a breakdown, was associated with PC in the present study. Indeed, it has been shown that children with enlarged tonsils tend to have more anterior and inferior position of the tongue.^30^ This lack of palatal support from the tongue causes harmful consequences, such as a narrow, short maxilla and a broader mandibular arch, with the consequent development of PC,^4^ which may explain the present results. It should be stressed that PC is often found in combination with AOB in the primary dentition,^29^ which may explain the association between AOB and not having undergone nose surgery. 

The absence of a normative diagnosis of oronasopharyngeal abnormalities and the consequent reliance on parental responses was the main limitation of the present study and may have led to an underestimation of the prevalence. However, the authors believe that the adequate educational level of the parents (> 8 years of study) contributed to a better concern with the child's health, thereby balancing this issue. It is worth mentioning that confounding variables, such as non-nutritive sucking habits, tong thrust and maxillofacial alterations,^13^ ^,^ ^22^ were not considered in the analysis and might have influenced the outcomes; which is a limitation of the study. Moreover, this was a population-based study in which children were randomly selected proportionally from all administrative districts of the city, and the results can therefore be extrapolated to the population. In view of the inherent limitations of the cross-sectional design, longitudinal studies and well-designed randomized controlled clinical trials should be carried out to determine the cause-and-effect relationship between oronasopharyngeal factors and the occurrence of malocclusions. 

## CONCLUSION

Significant associations were found between oronasopharyngeal-reported abnormalities and the presence of anterior open bite and posterior crossbite in preschool children.
